# Cell-specific occupancy of an extended repertoire of CREM and CREB binding loci in male germ cells

**DOI:** 10.1186/1471-2164-11-530

**Published:** 2010-09-29

**Authors:** Igor Martianov, Mohamed-Amin Choukrallah, Arnaud Krebs, Tao Ye, Stephanie Legras, Erikjan Rijkers, Wilfred Van Ijcken, Bernard Jost, Paolo Sassone-Corsi, Irwin Davidson

**Affiliations:** 1 Institut de Génétique et de Biologie Moléculaire et Cellulaire, CNRS/INSERM/UDS, 1 Rue Laurent Fries, 67404 Illkirch Cédex., France; 2 Erasmus Medical Center, Dr Molewaterplein 50, 3015GE Rotterdam, the Netherlands; 3 Department of Pharmacology, 2115 Gillespie Neuroscience, University of California, Irvine, California 92697-4625, USA

## Abstract

**Background:**

CREB and CREM are closely related factors that regulate transcription in response to various stress, metabolic and developmental signals. The CREMτ activator isoform is selectively expressed in haploid spermatids and plays an essential role in murine spermiogenesis.

**Results:**

We have used chromatin immunoprecipitation coupled to sequencing (ChIP-seq) to map CREM and CREB target loci in round spermatids from adult mouse testis and spermatogonia derived GC1-spg cells respectively. We identify more than 9000 genomic loci most of which are cell-specifically occupied. Despite the fact that round spermatids correspond to a highly specialised differentiated state, our results show that they have a remarkably accessible chromatin environment as CREM occupies more than 6700 target loci corresponding not only to the promoters of genes selectively expressed in spermiogenesis, but also of genes involved in functions specific to other cell types. The expression of only a small subset of these target genes are affected in the round spermatids of CREM knockout animals. We also identify a set of intergenic binding loci some of which are associated with H3K4 trimethylation and elongating RNA polymerase II suggesting the existence of novel CREB and CREM regulated transcripts.

**Conclusions:**

We demonstrate that CREM and CREB occupy a large number of promoters in highly cell specific manner. This is the first study of CREM target promoters directly in a physiologically relevant tissue *in vivo *and represents the most comprehensive experimental analysis of CREB/CREM regulatory potential to date.

## Background

Cyclic AMP response element (CRE) binding protein (CREB) and cyclic AMP response element modulator (CREM) are highly related bZIP proteins that regulate transcription in response to various stress, metabolic and developmental signals [1,2]. CREB and CREM bind to the consensus palindromic CRE 5'-TGACGTCA-3' or half-CRE 5'-TGACG-3' and 5'-CGTCA-3' elements present in the promoters of target genes. In somatic tissues, CREB activates transcription following phosphorylation of a serine residue (S133 in CREB, S117 in CREM) in the activation domain by several kinases and in response to a variety of stimuli [3]. Serine phosphorylation results in recruitment of the p300 and CREB binding protein (CBP) coactivators with histone acetyl-transferase activities [4]. Transcription regulation by CREB is also modulated by TORC (transducers of regulated CREB activity) 1 and 2 that interact with the bZIP DNA binding domain independently of serine 133 phosphorylation and modulate CREB interaction with the TAF4 subunit of TFIID, thus potentiating transcription activation [5]. TORCs play critical roles in several physiological processes such as glucose metabolism and obesity [6-9].

Multiple isoforms of CREM have been described that act either as repressors or activators [10]. Follicle stimulating hormone (FSH), by an as yet undefined mechanism, modulates the usage of alternative polyadenylation sites in mouse male germ cells, such that several destabiliser signals in the 3' untranslated region of the CREMτ activator isoform mRNA are eliminated leading to increased stability and the accumulation of the CREMτ protein to high levels in post-meiotic round spermatids [11,12]. In these cells, CREM bypasses the requirement for phosphorylation through interaction with the LIM-only domain protein ACT (activator of CREM in testis) encoded by the *Fhl5 *gene [13] specifically expressed in haploid cells. Knock out of CREM in mice leads to male sterility due to apoptosis of the round spermatids showing that it plays an essential role in spermiogenesis [14-16]. While loss of CREM leads to apoptosis of round spermatids and a complete arrest of spermiogenesis, only a handfull of CREM target genes have been described.

Here we have used chromatin immunoprecipitation coupled to massive parallel sequencing (ChIP-seq) to identify CREM target genes in round spermatids in testis and CREB target genes in spermatogonia derived GC1-spg cells. We identify more than 9000 genomic loci most of which are cell-specifically bound. Of these, more than 6700 target loci are occupied by CREM in testis, yet the expression of only a small subset of these genes are affected upon CREM knock out. We also identify intergenic CREB and CREM binding loci some of which are associated with H3K4 trimethylation suggesting the existence of novel CREB and CREM regulated transcripts.

## Results

### CREM binding sites in round spermatids of mouse testis

**Figure 1:**
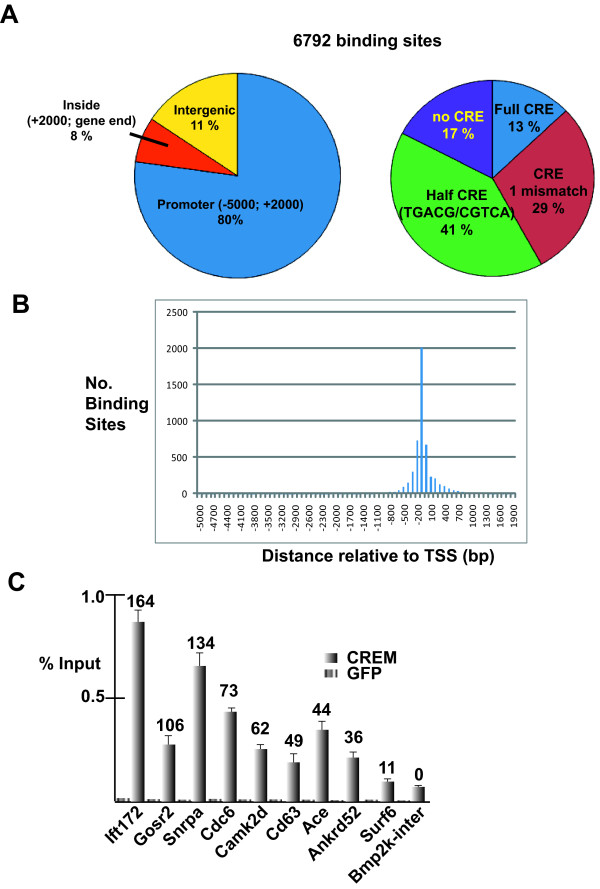
**Summary of CREM Chip-seq data in haploid cells**. **A**. Left hand pie chart shows genomic annotation of CREM binding sites with respect to known RefSeq genes. Promoter associated sites are arbitrarily confined to the regions between -5000 and +2000 bp with respect to the transcription start sites (TSS). The right hand pie chart indicates the frequency of occurrence of consensus or mutated CRE and half CREs at the CREM binding sites. **B**. Frequency of binding site localisation with respect to TSS. **C**. CREM ChIP-qPCR on the indicated loci. The GFP ChIP-qPCR is shown as control. Results are from three independent ChIP experiments. In panel C the numbers above each column represent the corresponding number of clustered reads in the ChIP seq experiments.

As previously reported, in testis, the CREMτ isoform is strongly and selectively expressed in round spermatids [12,15], while CREB is expressed in spermatogonia, Sertoli, Leydig and intertubular cells, but not in round spermatids (Additional File 1, Fig. S1A and B). To identify CREMτ binding sites, we performed duplicate ChIP-seq using anti-CREMτ antibody, or anti-GFP antibody as control, and formaldeyde-fixed testis chromatin from adult mice. We reproducibly identified around 6792 CREM-bound genomic loci (Fig. 1A and 1B, Additional file 2, Fig. S2, Additional file 3, Table S1). Of these loci, 80% were located in the promoters of annotated RefSeq genes, but a significant number were also located in intragenic regions corresponding to potential alternate promoters, and in intergenic regions with no annotated RefSeq transcripts (see below). Amongst the CREM targets are 46 miRNAs, some of which have been described as having a brain-specific function (Additional file 4, Table S2 and see below).

We used ChIP-qPCR to validate CREM binding to sites that showed high, medium and low occupancy in the ChIP-seq experiments. We compared CREM ChIP to control ChIP with anti-GFP antibody at a series of identified binding sites and with an intragenic region from the bone morphogenic 2 inducible kinase (*Bmp2k*) gene as a negative control and the testis-specific angiotensin I converting enzyme (*Ace*) promoter [17] as a positive control (Fig. 1C). These experiments confirmed binding of CREM to the tested promoters compared to the *Bmp2k *control. Strong occupancy of the intraflagellar transport 172 homolog (*Ift172*) and small nuclear ribonucleoprotein polypeptide A (*Snrpa*) loci was observed, but no significant occupancy of the surfeit gene 6 (*Surf6*) locus was seen, when compared to the background of the anti-CREM antibody as defined by the signal seen using the *Bmp2k *control. All loci defined by more than 40 clustered reads were significantly enriched in qPCR, not all loci defined by under 40 reads showed enrichment. This number was therefore used as a cut off to obtain a high confidence list.

Bioinformatic analysis of the binding sites showed that only 13% contained a full consensus CRE element, the majority contained either a CRE with one mismatch or one or several half-CRE elements (Fig. 1A). We also identified a significant fraction of sites where no CRE (17%) or half CRE (41%) could be observed. To determine whether an additional conserved sequence motif could be identified in these sites, the 100 most highly occupied regions were analysed using the Meme programme [18]. This analysis showed that the only frequently occuring motif corresponded to the SP1 binding site. No CRE or CRE related motif could be detected in this way and no novel alternative CREM binding motif could be identified. Loci containing full CREs generally showed a higher occupancy as 90% of these sites were defined by more than 100 clustered reads, while only 5% of the no CRE class fell into this category. Analysis of the location of CREM binding sites with respect to the transcription start site (TSS) showed a very strong preference around -150 bp irrespective of the presence of a CRE-related element (Fig. 1B).

To make a global correlation between promoter occupancy by CREM and transcription, we performed ChIP-seq using an antibody against trimethylated lysine 4 of histone H3 (H3K4me3). Trimethylation of this residue is tightly associated with transcription activity, a high density of this mark overlapping the 5'end of the gene indicates either its transcription or the presence of a poised preinitiation complex [19]. Comparison with the CREM ChIP-seq data set indicates that 95% of CREM bound promoters (ie with CREM occupied loci between -1000 and +500 bp with respect to the TSS) show H3K4me3. (Additional file 3, Table S1). However, in this experiment we cannot exclude that some genes are inactive in round spermatids where CREM is bound, but are transcribed at other developmental stages or in other cell types within the testis. To make a more accurate comparison we compared CREM promoter occupancy, H3K4me3 and gene expression using a transcriptome data set of adult mouse spermatid gene expression [20]. We identified 4934 CREM-occupied promoters showing H3K4 trimethylation. Comparison with array data showed that, while the 41% of probe sets on the array showed expression values ≥51, these accounted for 84% of the CREM and H3K4me3 associated genes (Additional file 5, Fig. S3A). We identified only 89 CREM occupied promoters (1.7% of the total CREM occupied proximal promoters) that were false negatives annotated as having no H3K4 trimethylation, but represented amongst the moderate to strongly expressed genes. These results indicate not only that the vast majority of CREM occupied promoters with H3K4 trimethylation are indeed expressed, but that they are over-represented amongst the higher expressed gene set. It should also be noted that we fail to detect any significant H3K4me3 of Leydig cell-specific genes such as *Cyp17a1 *and *Star *and little or no signal was seen for the *Rhox *genes that are specific for Sertoli cells. Thus, in agreement with the above data, the contribution of these cell types to the profile is negligible, indicating that the majority of the signal comes from transcription of genes in the germ cell lineage.

### Differing repertoires of CREM and CREB binding sites in round spermatids and spermatogonia GC1-spg cells

**Figure 2:**
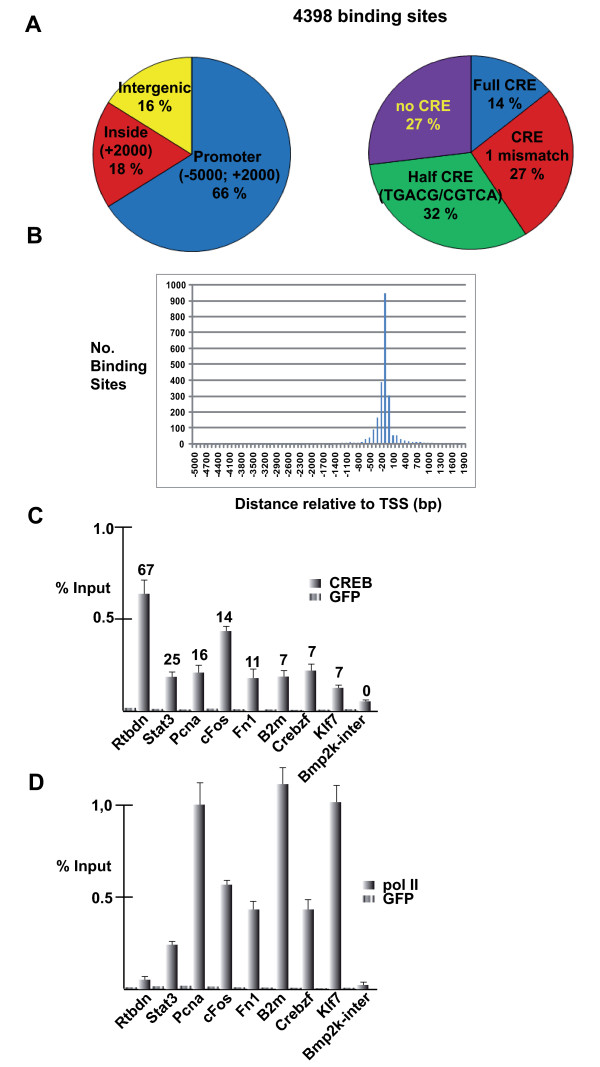
**Summary of CREB Chip-seq data in GC1-spg cells**. **A-D**. The layout is as described in Fig. 1.

As mentioned above CREB is not expressed in round spermatids, but is expressed in spermatogonia and in the somatic cells in testis. Due to its expression in several of the cell types that make up the testis, we cannot use total testis chromatin, even from early post-natal animals, for the ChIP-seq as we would be unable to distinguish in which cells CREB was occupying a given promoter. We therefore used GC1-spg cells that are immortalised spermatogonia [21] and express CREB, but not the CREMτ activator form (Additional file 1, Fig. S1B). ChIP-seq experiments in these cells using anti-CREB antibody allowed the identification of around 4398 binding sites (Fig. 2A, Additional file 2, Fig. S2 and Additional file 6, Table S3).

These results were confirmed by ChIP-qPCR where we observed significant, but variable occupation of all of the tested promoters. In this case even loci with small numbers of clustered reads showed significant enrichment with respect to the Bmp2k control (Fig. 2C). The majority of these promoters were also occupied by pol II (Fig. 2D).

Similar to what was observed with CREM, most CREB binding sites were observed in the promoters of annotated genes, but a signficant number of intra- and intergenic sites were also observed. Bioinformatic analysis showed that only a small number comprise full cannonical CREs and we identify a subset of sites with half-CRE or no identifiable CRE (Fig. 2A). Also similar to CREM, CREB binding sites are preferentially located around -150 relative to the TSS (Fig. 2B).

**Figure 3:**
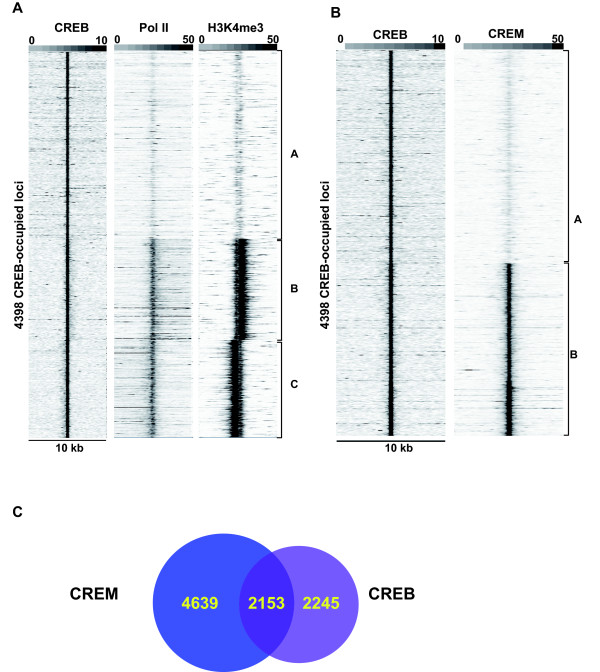
**Correlative analysis of CREB and CREM binding site occupancy**. **A. **Association of CREB, pol II and H3K4me3 in GC1-spg cells. Comparison of pol II and H3K4me3 tag density in the region of +/- 5 kb around the CREB-occupied loci. Clustering analysis identifies 3 classes, A; CREB loci with low or no pol II and H3K4me3, B-C; CREB loci with high pol II and H3K4me3 corresponding to transcription on the sense and anti-sense strands, respectively. **B. **Comparison of CREM tag density in the region of +/- 5 kb around the CREB-occupied loci. Clustering identifies 2 groups A; CREB loci with low or no CREM and B; CREB loci with high CREM. **C**. Venn diagramme comparing common and cell-specific CREB/CREM occupancy of binding sites using the CREB data in panel B and the equivalent analysis of the CREM occupied loci (data not shown).

To assess the association of CREB loci occupancy with transcriptional activity, we further performed H3K4me3 and pol II ChIP-seq in GC1-spg cells. Comparison with CREB occupancy shows that around 60% of CREB-occupied loci are associated with high levels of pol II and H3K4me3 in these cells (Fig. 3A, clusters B and C), while a second group shows little or no pol II and H3K4me3 (Fig. 3A, cluster A and see also Additional file 6, Table S3). Comparison with publically available array data for GC1-spg cell gene expression (GSE19355) showed that similar to what was observed in round spermatids, CREB and H3K4me3 occupied promoters were strongly over-represented in the highly expressed gene set (Additional file 5, Fig. S3B).

We next compared the repertoires of CREB and CREM binding sites in round spermatids and in GC1-spg cells. To identify cell-specifically occupied sites, we took the coordinates of the CREB binding sites and determined the read density in the CREM data set in a window of +/- 5 kb around these coordinates. If CREM and CREB occupy the same sites, there will be a high density of reads at these coordinates in both data sets. This comparison shows that a subset of loci show high occupancy in both cell types (Fig. 3B, cluster B), but the majority are cell-specifically occupied in either GC1-spg cells (Fig. 3B, cluster A) or in round spermatids (data not shown). In particular, CREM occupies a large set of sites in the round spermatids that are not occupied by CREB in GC1-spg cells.

**Figure 4:**
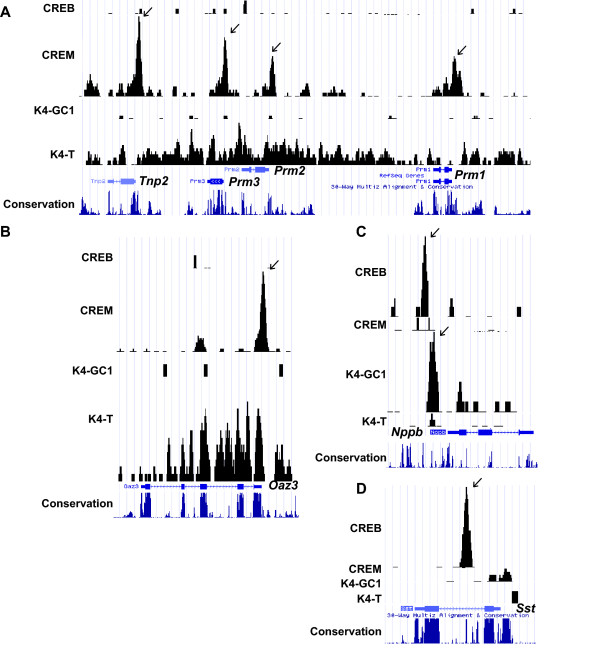
**Specific occupancy of target promoters by CREM and CREB**. **A-D**. Graphic representation of ChIP-seq results as. wig format files in UCSC web browser at the indicated loci. The same format has been used for Figs. 6 and 7 as well as Additional file 1, Figs. S4 and 5. The ChIP-seq results for CREB and CREM in GC1-spg and testis haploid cells respectively are shown along with results for H3K4me3 in GC1-spg cells (K4-GC1) and testis (K4-T). In addition, the mammalian conservation track from the UCSC browser is also included. CREM and CREB binding peaks are indicated by arrows.

For example, the genes encoding protamines 1, 2, and 3 and transition protein 2 are all selectively expressed in round spermatids and clustered on chromosome 16. Multiple binding sites are occupied by CREM at this locus in round spermatids, but not by CREB in GC1-spg cells and the locus shows H3K4 trimethylation only in testis (Fig. 4A). Similarly, the ornithine decarboxylase antizyme 3 (*Oaz3*) is occupied by CREM and active in testis, but not by CREB in GC1-spg cells, while the opposite is observed for the natriuretic peptide precursor type B (*Nppb*) locus (Fig. 4B and 4C). We also investigated occupancy of the well characterised CRE in the somatostatin (*Sst*) promoter [22]. *Sst *is not expressed in testis or in GC1-spg cells as shown by the absence of H3K4me3 and pol II (data not shown), and the promoter CRE is not occupied in either cell type. However, CREB selectively occupies an alternative and conserved intragenic site in GC1-spg cells (Fig. 4D). These results show that CREM and CREB occupy distinct but overlapping repertoires of binding loci in round spermatids and GC1 cells and that genes specifically activated in round spermatids are in general not occupied in GC1 cells, but become accessible during germ cell development in vivo.

In total, 9037 distinct CREB and CREM binding loci can be identified combining the ChIP-seq in round spermatids and GC1-spg cells. Despite the wide occupancy of potential binding sites by CREM in haploid cells, not all possible sites (i.e. full CREs) are occupied as the CRE element in the *Mitf-m *promoter that is specifically active in the melanocyte lineage, or the *Hoxa5 *gene are not occupied in GC1-spg or haploid cells (data not shown). These results show the existence of different classes of binding sites, those that are rather generally accessible, and a larger set that are occupied in a cell type-specific manner.

### Genes directly regulated by CREM in haploid cells

**Figure 5:**
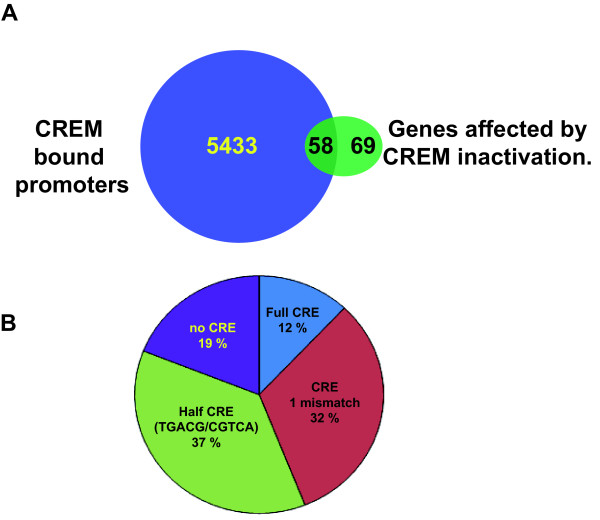
**CREM bound genes that are deregulated upon CREM inactivation in testis**. **A**. Venn diagramme comparing the total number of genes with CREM binding in their promoters with the genes deregulated in the Affymetrix analysis of Beisbarth et al. **B **Pie chart indicating the frequency of occurrence of consensus or mutated CRE and half CREs at the CREM binding sites in the promoters of the 58 deregulated genes.

CREM inactivation leads to round spermatid apoptosis at an early stage. To understand the basis of this effect, Beissbarth et al [23] used subtractive hybridisation and Affymetrix arrays to identify genes that are deregulated upon CREM inactivation. We used the results of the Affymetrix analysis to identify those genes whose promoters are bound by CREM and are direct targets. A set of 127 genes were reported to be deregulated, 102 of which are down-regulated, in CREM mutant testis [23] (http://www.dkfz.de/tbi_old/crem/cremgenes.txt). Of these, only 58 are direct CREM targets with one or several CREM binding sites in their promoters. (Fig. 5A and Additional file 7, Table S4). Surprisingly however, two of these *Rundc3a *and *Cnn1 *do not show significant H3K4me3 and are thus likely not expressed in the testis of the animals used in our experiments.

Analysis of the CREM binding sites in the promoters of regulated target genes shows that not all have consensus CRE elements, the profile is in fact similar to that seen in the global population of target genes, and some of the directly regulated genes do not contain any identifiable CRE or half CRE elements (Fig. 5B). It has previously been suggested that the presence of a TATA element is required for a gene to respond to cAMP stimulation via CREB [24], however, of the 58 CREM regulated promoters, only 6 have a consensus TATA element. This frequency is close to that seen in the genome as a whole [25]. Thus the presence of a TATA element is not a pre-requisite for regulation by CREM and is not enriched in CREM regulated promoters. Indeed many other CREM bound promoters have TATA elements and yet are not de-regulated in the absence of CREM.

The above results show that only 1% of the total occupied promoters are actually regulated by CREM in haploid cells. The expression of the vast majority of occupied genes is not affected by loss of CREM.

### CREB/CREM target promoters involved in other physiological processes

In addition to germ cell development, CREM and CREB are involved in many other physiological processes. We examined whether they occupy the promoters of target genes involved in other cell-specific functions. For example, the *Clock *and Period (*Per*) 2 genes are expressed in both testis and GC1-spg cells, yet their promoters are specifically occupied only in round spermatids by CREM (Additional file 8, Fig. S4A and B). Thus, while the expression of these genes is not haploid cell-specific, the CREB/CREM binding sites of their promoters are selectively occupied in germ cells. Moreover although not regulated by CREM in testis (see below), *Per2 *expression is for example regulated by CREB in response to parathyroid hormone activation of the cAMP signalling pathway in chondrocytes [26]. Thus at least in this case, the CREM promoter occupancy is indicative of potential regulation in other cell types by physiological signalling pathways.

**Figure 6:**
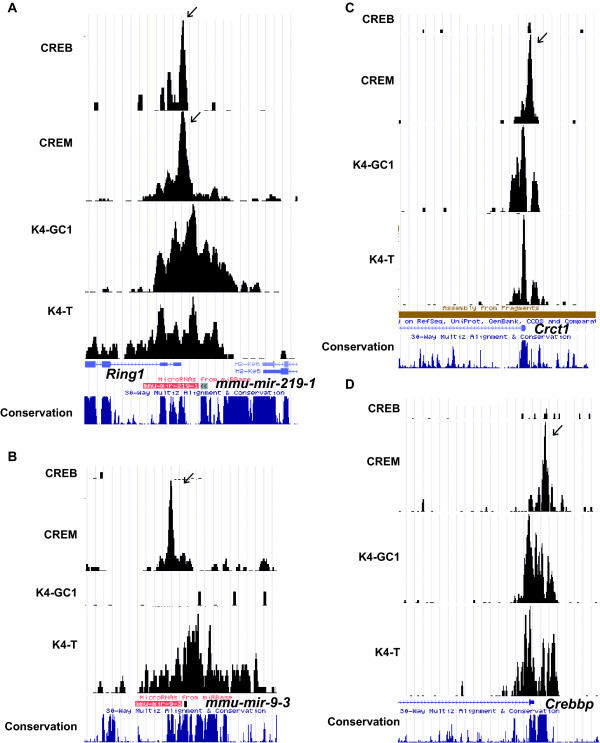
**Binding of CREB and CREM to the promoters of brain enriched miRNAs in haploid and GC1-spg cells or to the promoters of the coactivator CBP (*Crebbp*) and TORC1 (*Crtc1*) genes**. **A-D**. Graphic representation of ChIP-seq results in UCSC web browser at the indicated loci using the nomenclature described in Fig. 4.

Mmu-mir-132 and mmu-mir-219-1 are two CREB regulated brain-enriched miRNAs that regulate circadian rythmn and in the case of mir-132, neuronal morphogenesis [27,28]. At the mmu-mir-219-1 locus, a CRE element located downstream of the miRNA and upstream of the *Ring1 *gene is occupied in both haploid and GC1-spg cells by CREM and CREB respectively (Fig. 6A, and Additional file 4, Table S2). Furthermore, an extended region of H3K4 trimethylation in testis and in GC1-spg cells is observed indicating expression of both the miRNA and the adjacent *Ring1*. A similar observation was made at the mmu-mir-132 locus (data not shown). Mmu-mir-9-3 is a miRNA involved in silencing the neuronal transcription factor REST [29]. In contrast to the mmu-mir-219-1 and mmu-mir-132 loci, the mmu-mir-9-3 locus is specifically bound by CREM and active in testis and not GC1-spg cells (Fig. 6B). Thus in testis and in GC1-spg cells, CREM and CREB occupy the promoters of genes that are involved in specific processes in other cell types, and that although expressed in testis play no known role in spermatogenesis.

As CREB/CREM are involved in many physiological and metabolic processes, we used the KEGG pathway function of DAVID annotation (http://david.abcc.ncifcrf.gov/) to make a more comprehensive identification of potential CREB/CREM target genes in physiological functions (see Additional file 9, Table S5). This analysis identified many potential target genes not only in cell cycle and proliferation, but also in metabolic functions such as insulin signalling.

We also noted that CREB and CREM occupy their own promoters and those of several of their essential cofactors. The complex CREM locus comprises several alternative promoters where CREM/CREB occupy a site upstream of the major transcript that is expressed in both testis and GC1-spg cells, although as indicated above, the CREMτ activator isoform is not present in these cells (Additional file 8, Fig. S4C). Weak H3K4me3 of two of the alternate promoters is also seen specifically in testis. The genes encoding the coactivators CBP (*Crebbp*), p300 (*Ep300*) and TORC1 (*Crtc1*) are expressed in GC1-spg cells and testis, but their promoters are occupied only in haploid cells by CREM (Fig. 6C and 6D). These results suggest that in the appropriate cell type or in response to the appropriate signal CREB or CREM may regulate expression of their coactivators (see also [30,31]).

### Novel transcripts and loci potentially regulated by CREM and CREB

**Figure 7:**
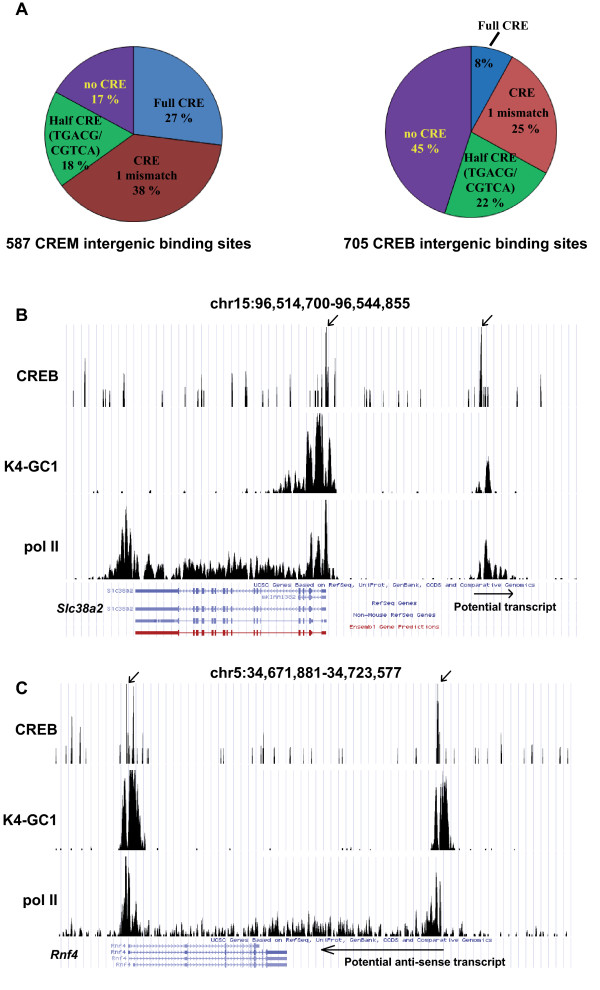
**Identification of novel CREB occupied regulatory elements and transcripts**. **A**. Pie chart indicating the frequency of occurrence of consensus or mutated CRE and half CREs at the CREM and CREB intergenic binding sites. **B-C**. Graphic representation of ChIP-seq results in UCSC web browser at the indicated loci using the nomenclature described in Fig. 4, with the addition of the GC1-spg pol II ChIP-seq data set. In panels B-C, the presence and direction of transcription of potential novel transcripts in the regions covered by elongating pol II are indicated by arrows.

It is striking that the majority of CREB and CREM binding sites are located at around -150 bp with respect to the TSS. The tight distance correlation between the CREB/CREM binding site and the transcription start site prompted us to investigate whether the CREB and CREM binding sites that are located in intergenic regions with no currently annotated RefSeq genes correspond to cryptic and as yet unidentified promoters of novel transcripts. Many of the intergenic sites are not associated with known RefSeq genes, but are associated with annotated EMBL transcripts or predicted genes most of which are not well characterised (See Additional files 3, Table S1 and Additional File 6, Table S3). However, there are also a set of loci with no nearby RefSeq annotation (Additional file 10, Table S6). Many of these loci are not associated with any predicted transcripts, but others lie close to UCSC gene predictions or Genbank transcripts corresponding to non-coding or uncharacterised RNAs. For CREM, 80% of these sites contain a CRE or half CRE, while for CREB, 55% contain CRE-related elements (Fig. 7A).

A subset of these regions potentially comprise active promoters as they are marked by H3K4me3. For example, on chromosome 15, downstream of the *Slc38a2 *gene, is a CREB occupied locus that correlates with a peak of H3K4me3 and pol II (Fig. 7B). The pol II is distributed as a peak close to the H3K4me3 peak marking the 5'end of the transcript and a lower density corresponding to the potential transcribed region. A similar pol II distribution is seen over the *Slc38a2 *gene where an additional peak is seen at the transcription termination site downstream of the gene, a profile typical of that previously observed for pol II [32,33]. This second region therefore appears to comprise an additional as yet uncharacterised transcript that can be potentially regulated by CREB. A similar example is seen on chromsome 5 at the *Rnf4 *locus where peaks of CREB and H3K4me3 and pol II are seen downstream of the *Rnf4 *gene that are characteristic of a promoter driving the expression of an anti-sense transcript with paused and elongating pol II. (Fig. 7C).

In other cases, the CREB occupied sites lie upstream of known promoters and are associated with H3K4me3, paused pol II, but little or no elongating pol II (Additional file 11, Fig S5A and B). These may correspond to alternative promoters that are not engaged in productive transcription, or distal enhancer elements. In contrast, other conserved loci show occupation by CREB and/or CREM, but low or no H34Kme3 and pol II. Again these may correspond to remote enhancer elements. Correlation between the CREM/CREB, H3K4me3 and pol II data sets thus allows the identification of potential CREB regulated novel transcripts, promoters, or distal enhancer elements.

In the course of this analysis we also noted multiple CREB binding sites and an exceptionally high RNA pol II density and over the region covering the MENε/β and Malat1 (metastasis associated in lung adenocarcinoma transcript 1) loci (Additional file 11, Fig. S5C). These correspond to two non-coding RNAs. The precise function of Malat1 is not known [34], while the MEN transcripts are involved in the formation of nuclear paraspeckles [35]. These two neighbouring loci have the highest pol II density observed in the data set and are thus very highly transcribed in GC1-spg cells.

## Discussion

### An extended repertoire of CREM/CREB binding sites

We have used ChIP-seq to identify CREM binding sites in round spermatids and CREB binding sites in GC1-spg cells. This is the first study of CREM target promoters directly in a physiologically relevant tissue *in vivo *and represents the most comprehensive experimental analysis of murine CREB/CREM regulatory potential to date. Our data identify 9037 binding loci that are occupied in either a common or cell-specific manner. The majority of binding sites cluster to a preferential location in the nucleosome free region around 150 nucleotides upstream of the TSS. This observation differs from a bioinformatic analysis showing that the CRE element is preferentially located around -45 bp relative to the TSS [36]. This discrepancy may be explained by the fact that many of the observed binding sites are half CREs, mutated or non-CRE elements, that many not have been identified bioinformatically. Thus, while full CREs may be closer to the TSS, the overall occupancy profile is centered around -150 bp. Moreover, a bioinformatics analysis of testis-specific promoters indicated a preferential presence of CRE-like elements rather than full consensus CREs [37]. This bias is also reflected in the experimental data.

Previous studies based on ChIP-chip analysis identified smaller repertoires of sites [30,38], while ChIP coupled to sequencing of concatenated tags in a SAGE type analysis identified more than 6300 CREB binding sites [31]. Based on this data, it has been suggested that there may be between 5000 and 19,000 binding sites in the human genome. Indeed, more than 10,000 full CREs have been predicted in the human genome of which around 4000 are suggested to be critical for function based on their conservation in mouse or the fact that they are present as a repeated cluster in promoter regions [30]. Our results show that in round spermatids around 622 full CREs are occupied while in GC1-spg cells around 613 CREs are occupied. Thus, many CREs are occupied neither in haploid cells nor in GC1-spg cells showing that the full repertoire of CREB/CREM binding sites exceeds the 9000 that we have identified.

From comparison of ChIP-chip in three different cell lines it has previously been suggested that CREB occupies almost the same set of target genes in each cell type, and that specificity of gene regulation is conferred not by selectivity in occupation of target sites, but by cooperation of CREB with other cellular factors [30]. In contrast, by ChIP and *in vivo *footprinting in hepatoma and PC12 cells or cortical neurons, Cha-Molstad et al., [39] showed that many sites were occupied in a cell specific manner. Our data clearly favour a model in which occupation of potential CREB/CREM binding sites is largely cell-specific although this does not exclude other criteria that specify which of the occupied sites are actually regulated by CREB and CREM. We have compared our murine data sets with those of Zhang et al obtained by ChIP-chip in human HEK cells [30]. We identified murine orthologues for 2003 of the CREB-occupied human promoters. Of these, 493 are occupied by CREB in GC1-spg cells, and 922 occupied by CREM in haploid cells. This comparison further highlights the cell-specificity of CREB/CREM promoter occupancy.

Cha-Molstad et al., also suggested that CREB preferentially occupies loci that are expressed and marked with H3K4 methylation. As an example, they show that occupancy of the well studied somatostatin (*Sst*) CRE correlates with its expression. In our experiments, the *Sst *locus is expressed neither in testis nor in GC1-spg cells and the promoter CRE is not occupied by CREB or CREM. In contrast, CREB occupies a novel and conserved intronic site in GC1-spg cells that does not contain any CRE-related element. However, a more global analysis of our CREB data set shows that a majority of CREB occupied promoters are also characterised by high pol II occupancy and H3K4 trimethylation. Thus the idea that CREB preferentially occupies promoters of expressed genes is generally valid. Similarly, CREM-occupied genes in haploid cells are over-represented amongst the highly expressed gene set On the other hand, there are both promoter and intergenic sites that are not closely associated with H3K4 trimethylation. Thus, expression is neither an absolute pre-requisite nor outcome of CREB/CREM occupancy. Moreover as discussed above, there are promoters that are active in both testis and GC1-spg cells, but bound only by CREM in testis. There must therefore be additional factors that influence occupancy, such as DNA methylation or competition with other factors whose binding sites overlap that of CREB [30,39].

### Insights into the specific role of CREM in spermiogenesis

During spermatogenesis, the gene regulatory programme in haploid cells is characterised by the activation of a large set of genes that are uniquely expressed in this cell type and by the existence of specialised mechanisms and factors that control their expression [40-42]. Given this specialisation, one could have imagined that in contrast to somatic cells, CREM would occupy a restricted set of loci corresponding to genes that are specifically expressed and regulated in these cells. Indeed at some loci, such as the protamine and transition protein genes, there is a developmental logic to CREM promoter occupancy. In contrast however, there are numerous examples of genes which are expressed in testis and GC1-spg cells, but are occupied only by CREM in haploid cells. Similarly, CREM also binds the promoters of genes that have no known or obvious function in spermatogenesis. Thus, contrary to what one may have expected for a highly specialised cell type with a unique gene expression programme, haploid cells clearly provide a much more permissive environment for accessibility than GC1-spg cells.

It has previously been shown that TBP, TFIIB, RNA polymerase II, and TFIIA are all strongly up-regulated in haploid spermatids [43,44]. It has been proposed that overexpression of the general transcription machinery at this stage is required to produce the large amounts of some mRNAs that have to be synthesised and stored for translation during the elongation and remodeling phase [43]. There is also evidence that overall mRNA levels are generally higher in haploid cells than in somatic cells due to 'promiscuous' transcription where in addition to transcription of highly expressed testis-specific genes and of normal 'housekeeping' genes, many other genes are also transcribed albeit at lower levels [43]. In agreement with this idea, our present ChIP-seq experiments show widespread occupancy by CREM of target genes that have no specific role in spermatogenesis, but whose promoters are active in haploid cells. The euchromatin of round spermatids is therefore highly accessible for transcription factor binding which, together with high levels of the basal transcription machinery, may lead to the expression of a large set of genes and generally higher mRNA levels.

Strikingly, only a very restricted set of CREM-occupied genes are affected by CREM inactivation. Comparison of the 127 genes whose expression is affected with those that are bound by CREM identifies 58 genes as direct targets. For this analysis, we used only the Affymetrix data of Beissbarth et al [23] that may underestimate the real number of affected genes. At the time of this study, many haploid expressed genes were not well characterised and were identfied only as ESTs. For example, we have previously characterised H1T2 (*H1fnt*) a histone H1 variant specifically expressed in haploid cells [45]. The *H1fnt *promoter is occupied by CREM, selectively activated in round spermatids and is therefore a direct CREM target. The full data set of Beissbarth et al actually comprises more than 367 regulated transcripts many of which are only identifed as ESTs. If the proportion of direct CREM targets from the Affymetrix data can be extended to the full set, this would suggest that CREM directly regulates between 150-200 genes in male germ cells. This number still represents only a small fraction of the CREM bound promoters. CREM clearly occupies many more promoters than it actually regulates. Neither the presence of a full CRE nor a TATA element are pre-requisites to predict whether a gene will be regulated by CREM. The criteria that determine whether a promoter will be regulated or not by CREM remain to be determined.

Inactivation of CREM leads to an early apoptosis of haploid cells around step 4 of their development [14,15]. Amongst the direct CREM targets are genes that encode protamines, transition proteins and outer dense fiber protein whose expression initiates around this stage, but whose gene products are required only later during the elongation stage. Reduced expression of these genes may account for the reduced sperm count and abnormalities seen in heterozygous CREM mutant mice, but do not explain the early round spermatid apoptosis. Previously, Beissbarth et al [23] suggested that down regulation of the anti-apoptotic genes *Bcl2 *and *Bcl6b *(BAZF) may contribute to germ cell apoptosis. *Bcl2 *is not a direct CREM occupied promoter (although the promoters of the related *Bcl2l1*, *Bcl2l11 *and *Bcl2l13 *genes are occupied by CREM), but *Bcl6b *is a direct target. Apoptosis may potentially result from direct deregulation of this gene or of one or several of the other direct CREM target genes involved in signal transduction and metabolic processes.

## Conclusions

Together our results have identified a large repertoire of CREM bound loci in haploid cells of which a small subset involved in signalling and metabolic processes are deregulated upon CREM inactivation and may explain the haploid cell apoptosis. In contrast to previous studies on smaller data sets, we have shown that promoter occupancy by CREB and CREM is highly cell specific in haploid and GC1 cells. This is the most comprehensive evaluation of CREB/CREM regulatory potential to date and we have identified a large set of promoters that may be regulated by these factors in different tissues and physiological processess. Correlation between the CREM/CREB, H3K4me3 and pol II data sets has identified many novel potential CREB regulated transcripts, and revealed the presence of CREB at novel start sites and distal regulatory elements.

## Methods

### ChIP-seq

Chromatin was prepared from the testes of 12 week old adult 129/SV mice. Animal breeding and manipulation were performed according to French national ethical standards using approved protocols. The animals were sacrificed and the testes removed and opened directly in 20 ml fixing solution (0.4% formaldehyde in 1× PBS) in a Petri dish. Seminiferous tubules were fixed for 10 minutes with agitation. The cross-linking reaction was stopped by adding 2 ml 2 M glycine solution. Seminiferous tubules were then centrifuged at 4000 rpm and the pellet was lysed with 2 ml cell lysis buffer (50 mM Tris pH8.0, 10 mM EDTA, 1% SDS) for 15 minutes. GC1-spg cells were obtained from the ATCC collection and cultured in DMEM with 10% foetal calf serum. Cells were grown to 90% confluency and crosslinked with formaldehyde 0.4% for 10 min at room temperature and the reaction was stopped by adding glycine to final concentration 0.2 M for 10 minutes at room temperature. Fixed cells were rinsed twice with PBS and resuspended in lysis buffer. Lysate from cells or testis was sonicated 30 min (10 sec on/30 sec off) in Diagenode water bath-sonicator and centrifuged at 14000 rpm for 10 min. The cleared supernatant was used immediately in ChIP experiments or stored at -80°C. ChIP was performed by standard procedures.

ChIP-seq was performed using an Illumina GAII sequencer and the raw data analysed by the Illumina Eland pipeline. Peak detection was performed using the MACS software (http://liulab.dfci.harvard.edu/MACS/) [46], and the peaks annotated using GPAT ([47], http://bips.u-strasbg.fr/GPAT/Gpat_home.html). Peak detection with MACS was performed using the GFP-ChIP as negative control. Clustering was performed by first generating density (.wig format files) counting the number of tags in a 25 pb sliding window for each ChIP-seq data set. The coordinates of the CREB binding sites were used as reference, and the tag density for the Pol II and H3K4me3 data sets were extracted from the. wig files in the 10 kb around the CREB site. A matrix of binding sites and densities was generated and subjected to K-means clustering using the Cluster 3.0 software (http://bonsai.ims.u-tokyo.ac.jp/~mdehoon/software/cluster/software.htm#ctv). The clustered matrix was visualized using Java TreeView. (http://jtreeview.sourceforge.net/).

Comparison of the ChIP-seq data with the array expression data was carried out by first performing an RMA normalisation of the mouse spermatid expression. Cel files from [20] and from the mock silenced GC1-spg data sets in the GEO data base (GSE19355). Excel files with the normalised data were compared to those comprising a list of CREM (or CREB) and H3K4me3-occupied promoters, thus assigning an expression value to each of the corresponding genes.

The following antibodies were used: the anti-CREMtau rabbit polyclonal antibody has been previously described [14,48], CREB (06-863, Upstate), H3K4me3 (MC315 04-745, Millipore), Pol II (H-224 Sc-9001 Santa Cruz). Real-time PCR was performed on a Roche Lightcycler using Roche SYBR Green mix. The following primer sequences were used. *Ift172*: 5'-TTTTGCCTCTCGTAGCACCT-3', 5'-ATGCAAACCTAACGCAAACC-3'

*Gosr2*: 5'-TAAAACTGGGAGGGATGCAG-3', 5'-CTAGGCCCATCTCATTTCCA-3'

*SnrpA*: 5'-CACAACGCTCTTGAAGGTCA-3', 5'-GGAACGGACCAATCAAAGAA-3'

*Cdc6*: 5'-AGACCTGGGGCTGTCCTATT-3', 5'-CCAAAGCCGCTCTACTTCAG-3' *Camk2d*: 5'-AAGCACGAGCACATAAGCAA-3', 5'-GCAGATTTAGAGGCCTACGG-3'

*Cd63*: 5'-ACCTGGTTTTGCCATCTCTG-3', 5'-AAGCCTTAGAGCTCCCTTGC-3'

*Ace*: 5'-GCTGGCACATTGCTCTATGA-3', 5'-AGCAGAGCAGCAGAAAGAGG-3'

*Ankrd52*: 5'-TGACGTCAGGGAGAGAGCTT-3', 5'-CCCCATCACAAGGAGAGAAA-3'

SURF6: 5'-GCCTTTCCCTTCATTTCCTC-3', 5'-GACCTGAGTATGGCGTGGAT-3'

Bmp2k intragenic: 5'-TCCCTGCACGTACTTTAGGC-3', 5'-TTGGAGCACTCAAAGGTGTG-3'

*Rtbdn*: 5'-TCCACGGTGCTGAAATATGA-3', 5'-CCAGGAACCGAGTTACAGGA-3'

*Stat3*: 5'-CTCCCCCACGCAATCTAGTA-3', 5'-AAGCATTTGGGTTTGTGGAG-3'

*Pcna*: 5'-GGGTTGGTAGTTGTCGCTGT-3', 5'-AGCACCTTCTTCAGGATGGA-3'

*Cfos*: 5'-TTAGGACATCTGCGTCAGCA-3', 5'-CCCGTCTTGGCATACATCTT-3'

*Fn1*: 5'-CCTCCCTTTCCTTCGAGTCT-3', 5'-GCCAGGTCTGGGACTAGAGA-3'

*B2m*: 5'-CTGACCGGCCTGTATGCTAT-3', 5'-TCCACCCTGTAGCCTCAAAG-3'

*Crebzf*: 5'-CCGGGTAGTACCGATGAGTG-3', 5'-CTTCGCTCCAGCCAGAGTAT-3'

*Klf7*: 5'-CCAGCGTGTACAGTGCAGAT-3', 5'-TGTCAGTGAGTTTGCGTTCC-3'

### Bioinformatics analysis

Detection of CRE and half CRE elements in the CREB and CREM bound loci was performed by first extracting a region of 500 nucleotides centered on the peak summit. These sequences were then analysed using the motif search programme of Genomatix software. Additional analysis of the bound regions was performed using the Meme programme (http://meme.nbcr.net/meme4_4_0/cgi-bin/meme.cgi) [18].

## Authors' contributions

IM designed and performed all of the ChIP assays, the immunostaining and immunoblot experiments, MAC, AK and TY developped and applied the algorithm for read density matrix clustering and AK also developped the ChIP-seq peak annotation programme, SL and ER performed the bioinformatics analyses of the ChIP-seq data, peak detection, motif detection, comparison with transcriptome data, WVI and BJ performed ChIP-seq library preparation and Illumina sequencing, PSC and ID concieved the project designed the experiments and ID wrote the manuscript. All authors have read and approved the final manuscript.

## Supplementary Material

Additional file 1 **Figure S1: Expression of the CREB and CREMτ proteins in adult mouse testis.A. **Immunofluoresence on sections from adult mouse testis with the anti-CREB and CREMτ antibodies. The signal for the antibodies in red, the Hoechst stained nuclei and the merged views are show as indicated. Representative examples of the different cell types are indicated by arrows. L; Leydig cells, S; Sertoli cells; Sp; spermatogonia; P; pachytene spermatocytes, I; intertubular cells, R; round spermatids, E; elongated spermatids. These results reveal a distinct expression of CREB and CREMτ in the testis, where CREB is present in the spermatogonia, the Sertoli, Leydig and intertubular cells, while CREMτ is present in haploid round spermatids. 20 fold magnification. **B**. Western blots with the CREMτ and CREB antibodies on 20 ug of extracts from the indicated cells or tissues. ES is undifferentiated mouse E14 embryonic stem cells, COS is COS-1, and 3T3 is NIH3T3. The CREB antibody detects a single polypeptide corresponding to CREB in each of the extracts, while the CREMτ antibody detects the CREMτ proteins only in the testis extract. Click here for file

Additional file 2 **Figure S2: Example of ChIP-seq data over an 11,191,448 bp region of mouse chromosome 3qA3-3qB.** The results for CREB and CREM in GC1 and haploid cells respectively are shown along with the ChIP-seq for H3K4me3 in GC1 cells (K4-GC1) and testis (K4-T). Representative CREB, CREM binding sites are indicated. Active promoters marked by H3K4me3 are also indicated. The * in the CREB and CREM chanels show examples of loci that are specifically occupied GC1 cells or haploid cells respectively. The * in the K4-GC1 and K4-T show examples promoters that are specifically or preferentially active in GC1 cells or testis, respectively. Click here for file

Additional file 3 **Table S1: CREM bound loci in mouse male haploid germ cells.** Excel table of annotated loci bound by CREM. Page 1 shows annotation of bound loci to RefSeq genes. N is number, Chr chromosome, start and end shows the coordinates of the binding sites and summit, the coordinates of the center of the site. The number of clustered reads and the pvalue and the local fold enrichment are indicated. For annotation, the RefSeq transcript ID, the gene symbol, and TSS coordinates are indicated along with the distance between the summit of the peak and the TSS. No match signifies that there was no RefSeq annotation within 5 kb from the peak summit position. The presence of a perfect CRE, a CRE with 0 or 1 mismatch or a half CRE are then indicated. The H3K4me3 status of the loci is also indicated. Page 2 shows annotation of bound loci to Ensembl transcripts. Layout is as in page 1 except that the RefSeq transcripts are replaced by Ensembl annotation. Click here for file

Additional file 4 **Table S2: MiRNAs that are targets of CREM in mouse male haploid germ cells.** Excel table of CREM bound miRNAs in haploid cells. Layout is essentially as in Table 1. Click here for file

Additional file 5 **Figure S3: Comparison of CREM and H3K4me3 ChIP-seq with adult mouse round spermatid gene expression.A**. The probe set expression values were divided into classes of ≤ 10, 11-50, 51-500 and ≥501 and the % of the total probe sets in each category are represented by the black bars as a % of the total. The % of the total number of CREM and H3K4me3 occupied promoters in each expression category is shown with the shaded bars. **B**. A similar representation is shown for the probe set expression values and CREB and H3K4me3 occupied promoters in GC1-spg cells. Click here for file

Additional file 6 **Table S3: CREB bound loci in GC1 cells.** Excel table of binding sites. Page 1 shows annotation of bound loci to RefSeq genes. Page 2 shows annotation of bound loci to Ensembl transcripts. Layout is as in Table S1. Click here for file

Additional file 7 **Table S4: Comparison of genes whose expression is affected by CREM inactivation in testis and CREM binding.** Page 1 of the Excel table reproduces the Affymetrix data of Beissbarth et al., (1) (http://www.dkfz.de/tbi_old/crem/cremgenes.txt) concerning genes whose expression is deregulated in the testis of CREM knock-out mice. Page 2 shows the data for CREM occupancy of the subset of loci that map to the promoters of the genes in page 1. Note that some promoters contain several CREM occupied sites; Page 3 shows the list of the genes whose expression is deregulated from page 1 and whose promoters are occupied by CREM. Click here for file

Additional file 8 **Figure S4: UCSC web browser graphic view of CREB and CREM binding to the indicated loci.A-B**. Selective binding of CREM to the *Clock *and *Per 2 *promoters. **C**. CREB, CREM occupancy and H3K4me3 over the *Crem *locus. Click here for file

Additional file 9 **Table S5: Functional annotation genes whose promoters are occupied by CREM (page 1) and CREB (page 2).** Functional annotation was performed using the KEGG pathway of David (http://david.abcc.ncifcrf.gov/). In addition to the list of genes in each identified functional category, are shown the number of genes (gene count) the % of the total number of genes, and pvalue. Click here for file

Additional file 10 **Table S6: Excel table of CREM and CREB bound sites with no annotation to RefSeq genes or Ensembl transcripts.** Pages 1 and 2 show the data concerning CREM and CREB binding sites for which no RefSeq annotation was found within 5 Kb of the peak summit and which were designated 'no match' in Tables S1 and S3. Layout is as in Table S1. Click here for file

Additional file 11 **Figure S5: A-B. UCSC web browser graphic view of CREB, H3K4me3 and pol II occupancy of intergenic sites upstream of annotated genes.** In panel A, no elongating pol II is seen between the upstream site and the annotated promoter, while in panel B, a low level of elongating pol II is observed, suggesting the existence of an alternative promoter. **C**. Multiple CREB binding sites and high pol II occupancy of the locus encoding the Malat1 and MENε/β non-coding RNAs. Click here for file
