# Supplementary figures and images for: Cell-specific occupancy of an extended repertoire of CREM and CREB binding loci in male germ cells
Source: BMC Genomics. 2010 Sep 29;11:530. doi: 10.1186/1471-2164-11-530 (PMC3091680; doi:10.1186/1471-2164-11-530)

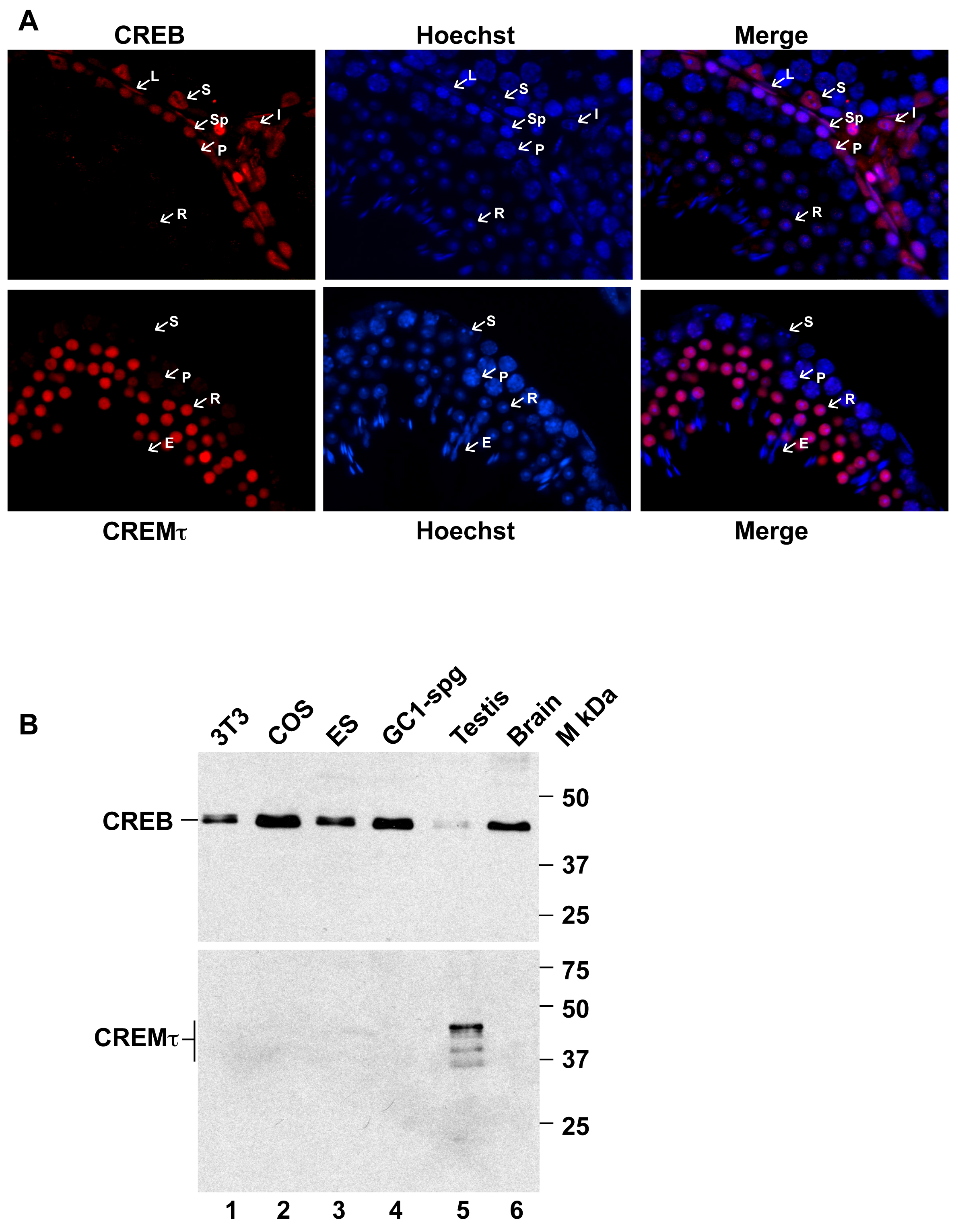

Supplement: Additional file 1 — Figure S1: Expression of the CREB and CREMτ proteins in adult mouse testis.A. Immunofluoresence on sections from adult mouse testis with the anti-CREB and CREMτ antibodies. The signal for the antibodies in red, the Hoechst stained nuclei and the merged views are show as indicated. Representative examples of the different cell types are indicated by arrows. L; Leydig cells, S; Sertoli cells; Sp; spermatogonia; P; pachytene spermatocytes, I; intertubular cells, R; round spermatids, E; elongated spermatids. These results reveal a distinct expression of CREB and CREMτ in the testis, where CREB is present in the spermatogonia, the Sertoli, Leydig and intertubular cells, while CREMτ is present in haploid round spermatids. 20 fold magnification. B. Western blots with the CREMτ and CREB antibodies on 20 ug of extracts from the indicated cells or tissues. ES is undifferentiated mouse E14 embryonic stem cells, COS is COS-1, and 3T3 is NIH3T3. The CREB antibody detects a single polypeptide corresponding to CREB in each of the extracts, while the CREMτ antibody detects the CREMτ proteins only in the testis extract. [file 1471-2164-11-530-S1.JPEG]

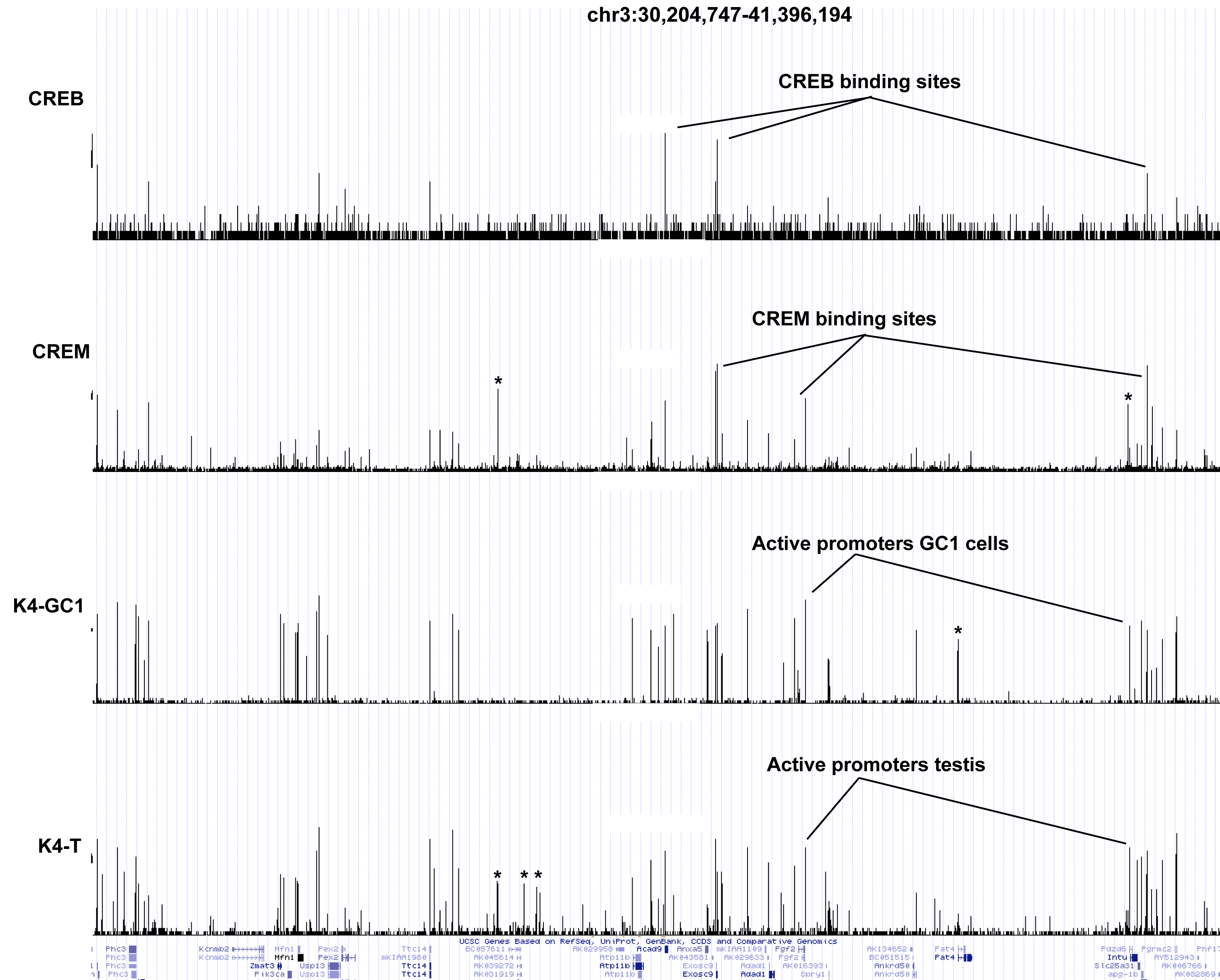

Supplement: Additional file 2 — Figure S2: Example of ChIP-seq data over an 11,191,448 bp region of mouse chromosome 3qA3-3qB. The results for CREB and CREM in GC1 and haploid cells respectively are shown along with the ChIP-seq for H3K4me3 in GC1 cells (K4-GC1) and testis (K4-T). Representative CREB, CREM binding sites are indicated. Active promoters marked by H3K4me3 are also indicated. The * in the CREB and CREM chanels show examples of loci that are specifically occupied GC1 cells or haploid cells respectively. The * in the K4-GC1 and K4-T show examples promoters that are specifically or preferentially active in GC1 cells or testis, respectively. [file 1471-2164-11-530-S2.JPEG]

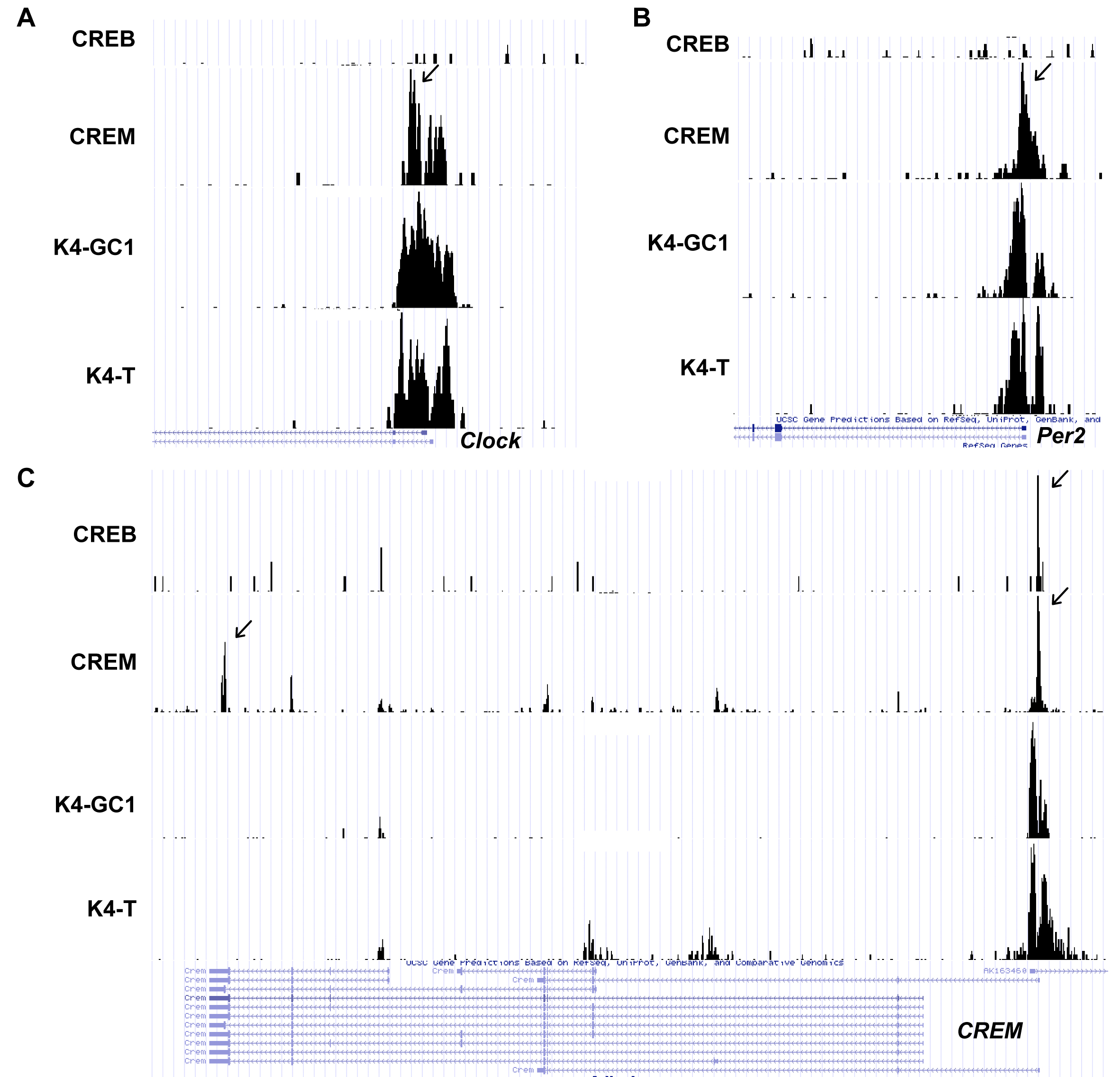

Supplement: Additional file 8 — Figure S4: UCSC web browser graphic view of CREB and CREM binding to the indicated loci.A-B. Selective binding of CREM to the Clock and Per 2 promoters. C. CREB, CREM occupancy and H3K4me3 over the Crem locus. [file 1471-2164-11-530-S8.JPEG]

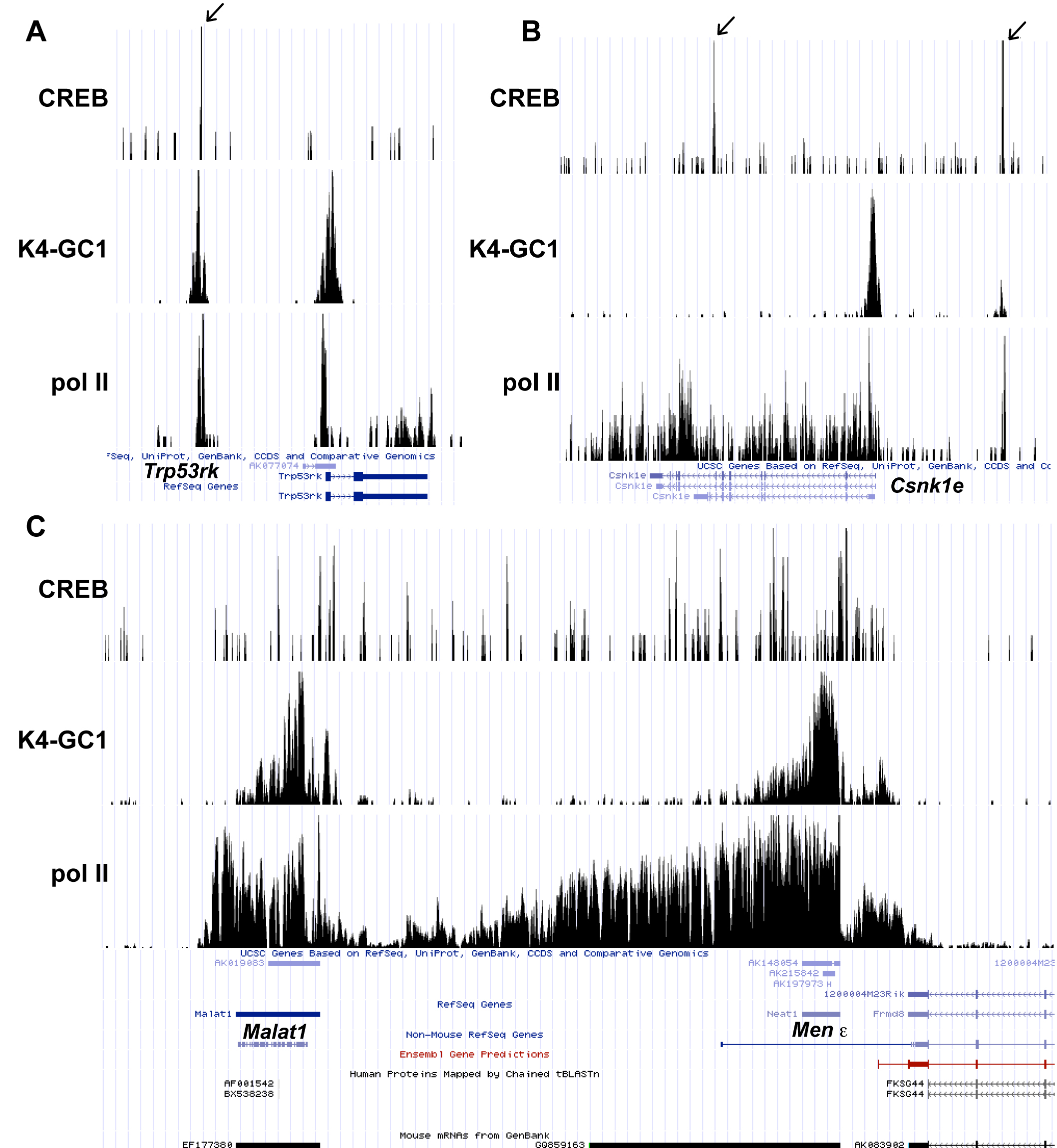

Supplement: Additional file 11 — Figure S5: A-B. UCSC web browser graphic view of CREB, H3K4me3 and pol II occupancy of intergenic sites upstream of annotated genes. In panel A, no elongating pol II is seen between the upstream site and the annotated promoter, while in panel B, a low level of elongating pol II is observed, suggesting the existence of an alternative promoter. C. Multiple CREB binding sites and high pol II occupancy of the locus encoding the Malat1 and MENε/β non-coding RNAs. [file 1471-2164-11-530-S11.JPEG]
