# Supplementary material for: Cell-specific occupancy of an extended repertoire of CREM and CREB binding loci in male germ cells
Source: BMC Genomics. 2010 Sep 29;11:530. doi: 10.1186/1471-2164-11-530 (PMC3091680; doi:10.1186/1471-2164-11-530)
Supplement: Additional file 5 — Figure S3: Comparison of CREM and H3K4me3 ChIP-seq with adult mouse round spermatid gene expression.A. The probe set expression values were divided into classes of ≤ 10, 11-50, 51-500 and ≥501 and the % of the total probe sets in each category are represented by the black bars as a % of the total. The % of the total number of CREM and H3K4me3 occupied promoters in each expression category is shown with the shaded bars. B. A similar representation is shown for the probe set expression values and CREB and H3K4me3 occupied promoters in GC1-spg cells. [file 1471-2164-11-530-S5.PDF]

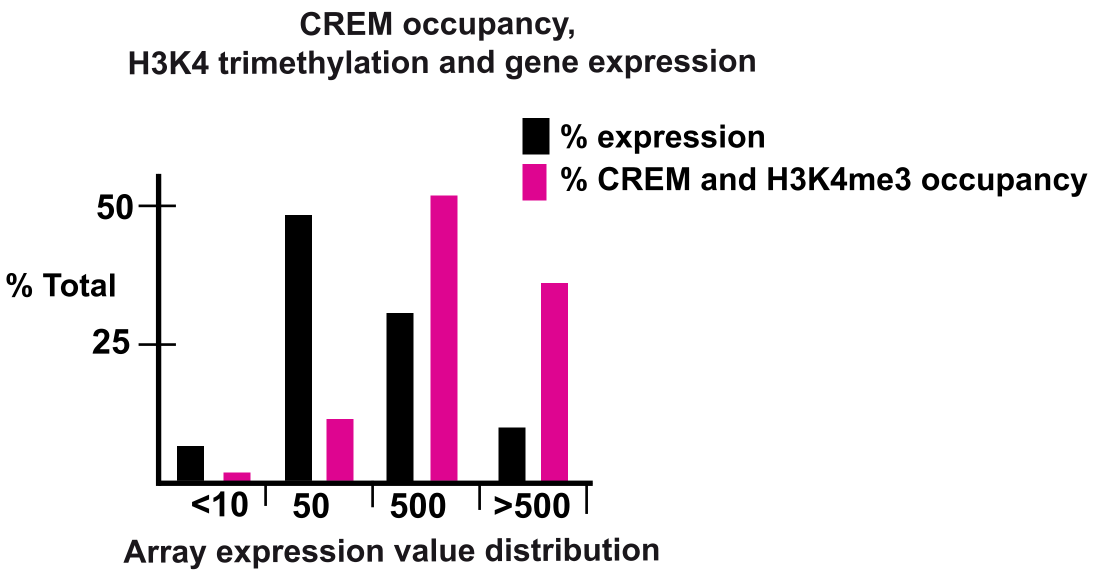
CREM occupancy,
H3K4 trimethylation and gene expression
% expression
% CREM and H3K4me3 occupancy
50
% Total
25
<10
50
500
>500
Array expression value distribution

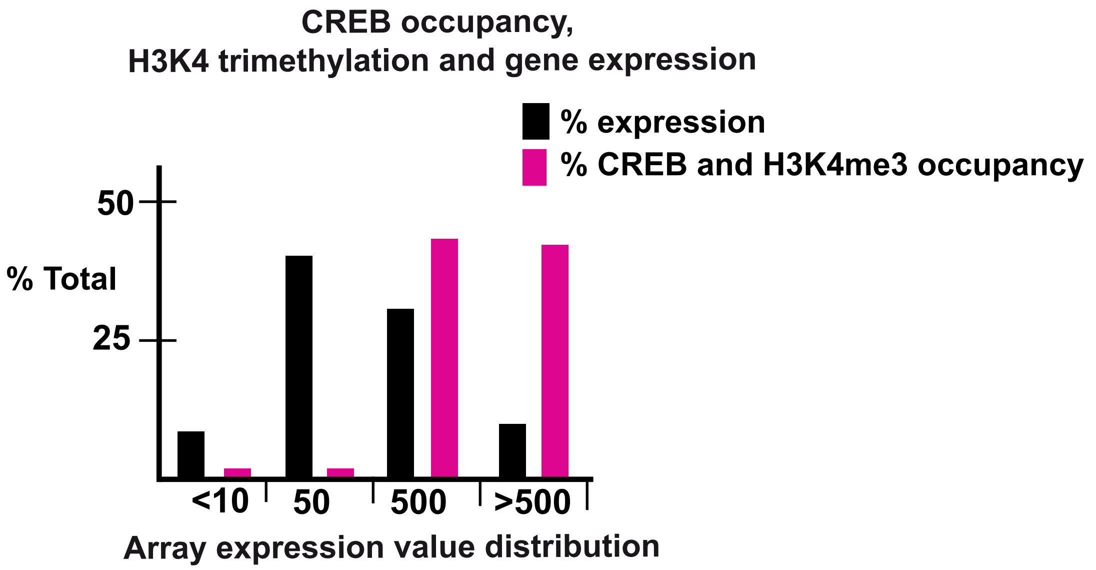
CREB occupancy,
H3K4 trimethylation and gene expression
% expression
% CREB and H3K4me3 occupancy
50
% Total
25
<10
50
500
>500
Array expression value distribution
